# Nonsteroidal sulfamate derivatives as new therapeutic approaches for Neurofibromatosis 2 (NF2)

**DOI:** 10.1186/s40360-019-0369-8

**Published:** 2019-11-15

**Authors:** Yu-chi Shen, Caroline Arellano-Garcia, Rosa E. Menjivar, Ethan M. Jewett, Wolfgang Dohle, Sofiia Karchugina, Jonathan Chernoff, Barry V. L. Potter, Kate F. Barald

**Affiliations:** 1 Department of Cell and Developmental Biology, 3029 BSRB, University of Michigan Medical School, Ann Arbor, Michigan 48109-2200 USA; 2 Department of Biomedical Engineering, College of Engineering, University of Michigan, Ann Arbor, Michigan 48109-2099 USA; 3 Present Address: Department of Human Genetics, University of Michigan, Ann Arbor, Michigan 48109-5619 USA; 4 NIH PREP program, Ann Arbor, Michigan 48109-5619 USA; 5 Present Address: Biology Graduate Program, Stanford University, Stanford, CA 94305 USA; 6 Cell and Molecular Biology Graduate Program, University of Michigan, Ann Arbor, MI 48109 USA; 7 Department of Electrical Engineering and Statistics, University of California, Berkeley, Berkeley, CA 94720-1770 USA; 8 Medicinal Chemistry & Drug Discovery, Department of Pharmacology, University of Oxford, Mansfield Road, Oxford, OX1 3QT UK; 9 Cancer Biology Program, Fox Chase Cancer Center, 333 Cottman Ave, Philadelphia, PA 19111 USA

**Keywords:** Neurofibromatosis 2, Nonsteroidal sulfamate derivatives, Tumour treatment, Cytoskeleton

## Abstract

**Background:**

Neurofibromatosis 1 and 2, although involving two different tumour suppressor genes (neurofibromin and merlin, respectively), are both cancer predisposition syndromes that disproportionately affect cells of neural crest origin. New therapeutic approaches for both NF1 and NF2 are badly needed. In promising previous work we demonstrated that two non-steroidal analogues of 2-methoxy-oestradiol (2ME2), STX3451(2-(3-bromo-4,5-dimethoxybenzyl)-7-methoxy-6-sulfamoyloxy-1,2,3,4-tetrahydroisoquinoline), and STX2895 (7-Ethyl-6-sulfamoyloxy-2-(3,4,5-trimethoxybenzyl)-1,2,3,4-tetrahydroisoquinoline) reduced tumour cell growth and induced apoptosis in malignant and benign human Neurofibromatosis 1 (NF1) tumour cells. In earlier NF1 mechanism of action studies we found that in addition to their effects on non-classical hormone-sensitive pathways, STX agents acted on the actin- and myosin-cytoskeleton, as well as PI3Kinase and MTOR signaling pathways. Tumour growth in NF2 cells is affected by different inhibitors from those affecting NF1 growth pathways: specifically, NF2 cells are affected by merlin-downstream pathway inhibitors. Because Merlin, the affected tumour suppressor gene in NF2, is also known to be involved in stabilizing membrane-cytoskeletal complexes, as well as in cell proliferation, and apoptosis, we looked for potentially common mechanisms of action in the agents’ effects on NF1 and NF2. We set out to determine whether STX agents could therefore also provide a prospective avenue for treatment of NF2.

**Methods:**

STX3451 and STX2895 were tested in dose-dependent studies for their effects on growth parameters of malignant and benign NF2 human tumour cell lines in vitro. The mechanisms of action of STX3451 and STX2895 were also analysed.

**Results:**

Although neither of the agents tested affected cell growth or apoptosis in the NF2 tumour cell lines tested through the same mechanisms by which they affect these parameters in NF1 tumour cell lines, both agents disrupted actin- and myosin-based cytoskeletal structures in NF2 cell lines, with subsequent effects on growth and cell death.

**Conclusions:**

Both STX3451 and STX2895 provide new approaches for inducing cell death and lowering tumour burden in NF2 as well as in NF1, which both have limited treatment options.

## Background

Both Neurofibromatosis 1 and 2 (NF1 and NF2) are disorders characterized by the formation of tumours of the peripheral and central nervous system [1], primarily affecting cells of neural crest origin [2]. Although other organ systems and cell types are affected in both NF1 and NF2, the cell of origin in most malignancies is the Schwann cell [1]. Both NF disorders arise through autosomal dominant inheritance with loss-of-function mutations in the tumour suppressing functions of the respective tumour suppressor genes: Neurofibromin (NF1) and Merlin (NF2) [3, 4].

Neurofibromatosis type II (NF2) is associated with loss-of-function mutations in the NF2 gene that encodes the multi-functional protein, Merlin (Moesin-Ezrin-Radixin-like protein) [5], also known as Schwannomin. Merlin is currently an out-group member of the ERM (Ezrin-Radixin-Moesin) protein family because it is the only one in the family to function as a tumour suppressor. Strong evidence suggests that Merlin regulates the assembly of apico-lateral junctional complex [6]. Merlin is also involved in stabilizing membrane-cytoskeletal complexes [7], in cell proliferation [8–10], and in apoptosis [10]. Conditional knockouts of Merlin result in the formation of meningiomas [11]. Conditional deletion of Merlin also contributes to hyperplasia of Schwann cells and of neural-crest derived odontoblasts, osteoblasts, and renal tubular cells. It also results in metastases of osteoscarcoma and fibrosarcoma [12]. Loss of Merlin activates several mitogenic pathways including Rac1/Pak [13, 14], Ras/Raf, PI3K/AKT, mTORC1 and Wnt/β-catenin pathways [15, 16]. Merlin also mediates the Hippo pathway and inhibits proliferation, acting in the nucleus to bind E3 ubiquitin ligase CRL4^DCAF1^ [17].

NF2 affects one in 25,000–30,000 live births worldwide. A hallmark of the disease is the formation of bilateral vestibular Schwannomas, as well as the formation of multiple meningiomas, extramedullary spinal tumours, and ependymomas [18]. Uncontrolled growth of these tumours can also lead to cataracts, hearing loss, balance issues and paralysis [5, 6, 19]. Although malignant transformations of NF2 tumours are rare, better therapeutics are needed, because numerous tumours can lead to early morbidity and early mortality (age 36) [5].

Current treatment options for NF2 tumours include surgical resection of either part of or the complete tumour, which is difficult to perform without damaging nerves. Stereotactic radiosurgery is also an option, however the risk of malignant transformation rises several years post-surgery [20, 21]. Alternate treatment options for NF2 tumours include inhibitors of the epidermal growth factor receptor (EGFR) [22], inhibitors of the vascular endothelial growth factor (VEG-F) [23–25], inhibitors of mTORC1 [26], an inhibitor of platelet-derived growth factor (PDGF) [27], and an inhibitor of histone deacetylase (HDAC) [28]. However, such treatments have resulted in mixed and sometimes limited success in human trials [29]. Current phase II clinical trials explore better treatment options through inhibition of the mTORC1, PDGF-R, VEGF and anti-angiogenic pathways (NCT01419639; NCT00561665; NCT00589784; NCT02104323). To date, no phase III clinical trials for the treatment of NF2-related disorders have been initiated.

Previous studies from our laboratories [30] demonstrated that sulfamate ester derivatives of a class of nonsteroidal tetrahydroisoquinoline (THIQ)-derived agents, derived by SAR translation from the naturally occurring anticancer metabolite of 17-β estradiol, known as 2-methoxyoestradiol (2ME2) [31, 32] are highly effective at reducing cell viability in hormone-responsive NF1 cell lines. These derivatives, known as STX3451 and STX2895, are capable of inducing apoptosis in cell lines derived from two malignant peripheral nerve sheath tumours (MPNST) at very low concentration [30].

**Fig. 1:**
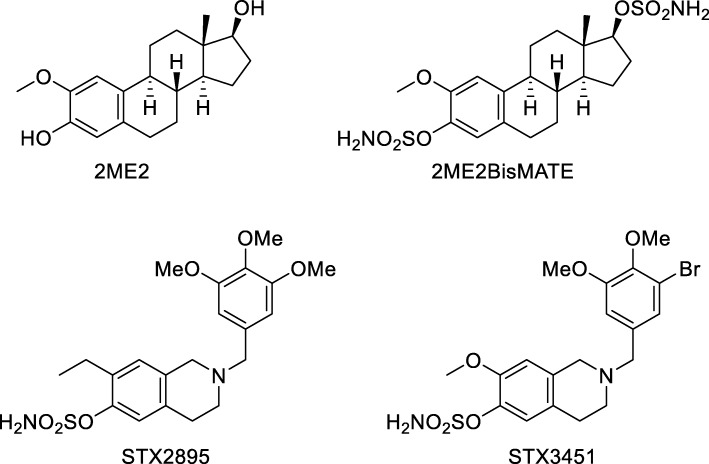
Chemical structures of 2-MeE2, 2ME2BisMATE, STX2895 and STX3451

The two non-steroidal 2ME2 analogues, STX3451 (2-(3-bromo-4,5-dimethoxybenzyl)-7-methoxy-6-sulfamoyloxy-1,2,3,4-tetrahydroisoquinoline) and STX2895 (7-Ethyl-6-sulfamoyloxy-2-(3,4,5-trimethoxybenzyl)-1,2,3,4-tetrahydroisoquinoline) (Fig. 1) were synthesized as previously described for treatment of a variety of hormone-responsive cancers, including breast cancer and prostate cancer [31–35]. STX3451 displays high anti-proliferative activity across the NCI 60 cell line panel (average GI50 40 nM) [31]. STX2895 also has a high anti-proliferative activity with GI_50_ of 40 and 52 nM on DU-145 human prostate and MDA MB-231 breast cancer cell lines, respectively [32]. The ability of STX3451and STX2895 to disrupt normal microtubule dynamics through competitive binding to the colchicine site, in a cell-free system, appears to make a significant contribution to their anti-proliferative effects [31, 32]. Sulfamoylated derivatives of 2ME2, particularly its bis-sulfamate 2ME2BisMATE, have been shown to be more effective, more bioavailable, resistant to catabolism in the gut and liver than 2ME2 itself [36], and also interact with carbonic anhydrase IX, a hypoxic tumour target [37]. Although the bioavailability of STX3451 and STX2895 has not been examined in an animal model, the in-vitro effects of these molecules on NF1 cell lines derived from both benign and malignant human tumours are particularly promising [30]. These agents are multi-faceted with multiple modes of action, including those that are hormone-independent ([30]). Recently, some of us also demonstrated that THIQ derivatives, including STX2895 and STX3451 exert anti-proliferative and antimitotic effects, induce apoptosis and involve autophagic processes in MDA-MB-231 metastatic breast and A549 epithelial lung carcinoma cell lines [38].

Here, we show that NF2 status plays a role in the effectiveness of these non-steroidal 2ME2 derivatives on cell viability in vitro. STX2895 and STX3451 treatment on NF2 null (−/−) cell lines induces nuclear fragmentation, microtubular disruption, inhibition of cellular migration/wound healing, and can induce apoptosis in both benign and malignant NF2 tumour-derived cells.

## Methods

### Cell culture

NF2-null malignant meningioma KT21-MG1-Luc5D cells (KT21 cells, derived from a human malignant meningioma cell line KT21-MG1 [39]) and the luciferase-expressing NF2-deficient benign meningioma cell line, Ben-Men-1-LucB (Ben-Men-1 cells, derived from a human benign meningioma cell line Ben-Men-1 [40]), have been described previously [41]. CH157MN cells (a human malignant meningioma NF2^−/−^ cell line from a 41-year-old woman [42]) were generously provided by Dr. Yancey Gillespie at the University of Alabama Birmingham. HEI-193 cells (a benign vestibular Schwannoma cell line from a 56-year-old Neurofibromatosis type 2 (NF2) patient [43]) were kindly provided by Dr. Xandra Breakefield, Massachusetts General Hospital. The IOMM-Lee (a malignant Meningioma NF2^+/+^ cell line derived from the brain of 61-year-old man [44]) was obtained from Dr. Randy Jensen at the University of Utah. BJ (ATCC® CRL-2522™, human foreskin fibroblast) cells were a gift from Dr. Sem Hin Phan, University of Michigan. Most cell lines were cultured in high-glucose Dulbecco’s Modified Eagle Medium (DMEM) (Gibco), supplemented with 10% fetal bovine serum (FBS) (Atlanta Biologicals, Flowery Branch, GA), 2 mM L-glutamine and 100 U/ml penicillin/streptomycin (Gibco), at 37 °C in a humidified 5% CO_2_ incubator [41]. HEI-193 cells were maintained in DMEM/10% FBS with forskolin (Cayman Chemical, Ann Arbor, MI), recombinant glial growth factor 2 (R&D Systems, Minneapolis, MN), and geneticin (Gibco). CH157MN cells were grown in MEM/F12 medium with 7% FBS.

## Compounds

STX3451 and STX2895 were synthesized according to previously published protocols [31, 45]. Pak inhibitors, Frax1036 and PF3758309 were used as control to be compared with the effects of STX3451 and STX2895 on NF2-deficient cells. Frax1036 was purchased from Afraxis Inc., San Diego, CA [39]. PF3758309 was synthesized according to published protocols [46].

### Cell viability assays

Cells were plated in 96-well Primaria™ plates (BD Falcon,1 × 10^4^/well) determined by a hemocytometer. STX3451 or STX2895 at concentrations of 100 nM, 300 nM, 600 nM, and 1000 nM was dissolved in vehicle (DMSO; Sigma-Aldrich, St Louis, MO, USA) and were added to the wells 2 hours after plating the cells. The final concentration of DMSO was 1%. PF3758309 (PF) and Frax1036 concentrations ranged from 0.03 μM to 20 μM and from 2.5 μM to 25 μM, respectively. The final concentration of DMSO was 2.5%. STX3451, STX2895, PF, or Frax1036 was replenished after 2 days of culture. Cell growth was assessed using CellTiter 96 (Promega, Madison, WI, USA) [30, 47, 48].

### Immunohistochemistry

40,000–80,000 cells plated on 0.1% porcine gel-coated coverslips were treated with DMSO, STX3451, STX2895, PF3758309, or Frax1036 for various time periods. Immunocytochemistry and counter-staining with DAPI (4′,6-diamidino-2-phenylindole, Molecular Probes, Eugene, OR), were carried out as reported previously [45]. Microtubules were visualized using antibodies for α-tubulin (1:500, Sigma). Apoptosis was assessed by an anti-annexin V antibody (1:500, Abcam ab14196). Fluorescent (Alexa Fluor 488 or 594) anti-rabbit or anti-mouse secondary antibodies (Life Technologies) were used for visualizing the labeled cells. Fluorescence micrographs were taken with an Olympus BX-51 microscope and processed with Adobe Photoshop.

### Wound-healing assays

The ability of cells to migrate was assessed by wound-healing assays [49, 50]. 2 × 10^5^ cells were plated in a 10 mm cell culture dish and allowed to grow to confluence. Wounds were made using a ruler to maintain a straight edge and a 200 μl pipette tip applied to the bottom of the dish to remove the cells. The culture plates were then washed twice with PBS. Fresh medium with DMSO, STX3451, or STX2895 was added to the dishes. STX3451 and STX2895 were used at 300 nM for KT21, Ben-Men-1, and HEI-193 cells, 1000 nM for IOMM-Lee and BJ cells. Photographs of the wounds and subsequent cell migration and “wound filling” were taken using a Nikon SMZ1500 dissecting microscope and digital camera (Nikon Digital Sight) at the time points indicated. The dimensions of the “wound” were measured with ImageJ software.

### Western blots

Cell lysates were collected after 24 h treatments with DMSO, STX3451, STX2895, PF, or Frax1036. Western blotting was performed as described in the studies on NF1 [30]. Membranes were incubated with antibodies (all from Cell Signaling Technology) to phosphorylated S6, S6, cyclin D, phosphorylated MEK, MEK, Pak 1/2, phospho-Pak1 (pPak1), phospho-Pak2 (pPak2) and GAPDH. The peroxidase-coupled secondary antibody (1:1000) and chemiluminescent HRP substrate (Millipore) were used to detect the labeled bands. Pixel density was quantified with ImageJ (NIH) software.

## Results

### The effects of nonsteroidal analogues of 2ME2BisMATE on cell viability depend on NF2 status

The STX therapeutic agents tested are multifaceted in their mechanisms of action. This study was directed at discovering whether the mechanisms of action that make the STX agents promising potential therapeutics for the treatment of NF1 would also apply to the potential treatment of NF2. Our previous studies concluded that the agents’ effects on the growth of and induction of apoptosis in human NF1 tumour cells were both through NF1-specific pathways and through significant effects on both the actin-based and myosin-based cytoskeleton [30]. The same approaches in the present study on NF2 led to the findings that, in NF2 human tumour cells, none of the pathways that were found to be affected in NF1 was affected in NF2. However, effects on NF2 cell growth were also mediated through the STX agents’ effects on the cytoskeleton.

**Fig. 2:**
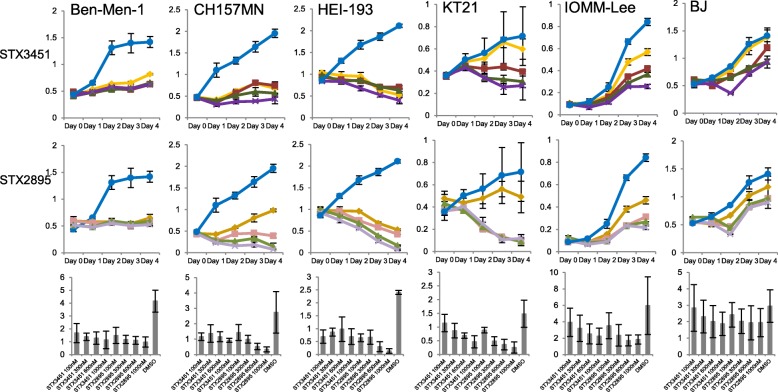
Cell viability of NF2-null and NF2-wild type tumour cells in response to the 2ME non-steroidal sulfamate derivatives STX3451 and STX2895. DMSO; STX3451 0.1 μM; STX3451 0.3 μM; STX3451 0.6 μM; STX3451 1 μM; STX2895 0.1 μM; STX2895 0.3 μM; STX2895 0.6 μM; STX2895 1 μM. Graphs show the increase in absorbance (fold change) after 4 days of treatment compared to the levels at the beginning of the treatment

In our earlier report [30] we demonstrated that relatively low concentrations of STX3451 have profound effects on the cell viability of NF1 tumour cell lines, even in the presence of elevated oestrogen and progesterone hormones. We also demonstrated that these effects were caused by more than one mechanism of action. To examine whether STX3451 or STX2895 affected the viability of human NF2 tumour cells, in a manner similar to those in NF1 tumour cells, we exposed Ben-Men-1(NF2^−/−^ benign meningioma cells), CH157MN (NF2^−/−^ meningioma cells), HEI-193 (NF2^−/−^ benign vestibular Schwannoma cells), KT21 (NF2^−/−^ malignant meningioma cells) and IOMM-Lee (NF2^+/+^ malignant meningioma cells) to the individual agents or to a combination of the two. Our results showed that both agents either completely blocked cell proliferation or induced cell death in NF2^−/−^ cells whether the cell lines were derived from benign or malignant tumours (Fig. 2). Cell proliferation of wild-type NF2 malignant meningioma IOMM-Lee cells was notably reduced when treated with either drug. In all but the CH157MN cell line, STX2895 was slightly more potent than STX3451. BJ fibroblast cells were seen to have reduced cell growth rate when treated with either compound, but cell viability was not reduced even with the highest concentrations of either drug we used (1 μM).

**Fig. 3:**
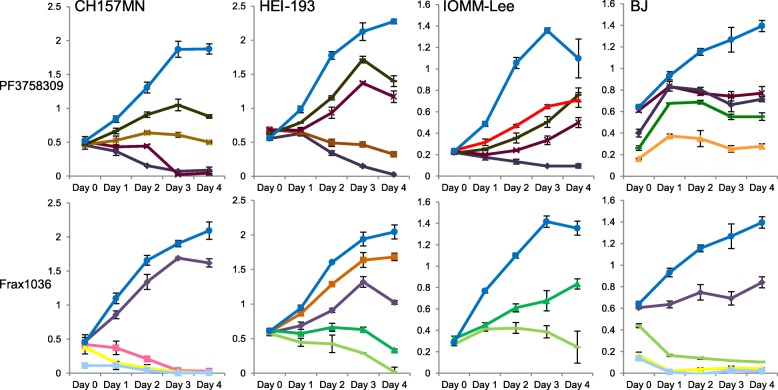
Cell viability of NF2-null and NF2-wild type tumour cells after treatment with the Pak inhibitors PF3758309 and Frax1036. DMSO; PF 0.03 μM; PF 0.1 μM; PF 0.5 μM; PF 1 μM; PF 2.5 μM; PF 5 μM; PF 10 μM; PF 20 μM; Frax1036 2.5 μM; Frax1036 5 μM; Frax1036 7.5 μM; Frax1036 10 μM; Frax1036 15 μM; Frax1036 20 μM; Frax1036 25 μM

Chow et al. had previously shown that Pak inhibitors reduced proliferation and survival of both Ben-Men-1 and KT21 cells [41]. We set out to examine the effects of such inhibitors on other NF2-null cells as well as CH157MN cells. Our results demonstrate that Frax1036 (a group I Pak-specific inhibitor) is less potent than the analogues of 2ME2BisMATE in promoting cell death of either CH157MN or HEI-193 cells (Fig. 3). STX3451 halted CH157MN cell growth completely at a concentration of 1 μM, whilst STX2895 induced cell death at 300 nM. By contrast, these cell lines still grew relatively rapidly in 5 μM Frax1036. Only at 15 μM did Frax1036 induce apoptotic cell death in CH157MN. PF3758309 (PF), an inhibitor for both groups I and II Paks, on the other hand, was found to be more potent than STX3451 but less potent than STX2895 for CH157MN cells. PF at 500 nM halted cell growth of CH157 MN cells (Fig. 3), whilst STX2895 and STX3451 had the same effect- halting CH157 MN cell growth- at 300 nM and 1000 nM, respectively (Fig. 2). Both STX3451 and STX2895 were very effective at low concentrations (100 nM) in promoting the death of HEI-193. However, neither PF nor Frax1036 proved to be as potent for reducing cell viability in benign vestibular Schwannoma HEI-193 cells. We did not test concentrations higher than 1 μM with the non-steroidal analogues for any of the cell lines, except for IOMM-Lee cells. PF3758309 began to cause cell death in IOMM-Lee cells between 1 and 5 μM, whilst the cells still grew – albeit at a lower rate – even at 7.5 μM Frax1036. BJ cells were less sensitive to PF and Frax1036 than any of the tumour cell lines. Even at 20 μM, PF did not reduce BJ cell viability, although BJ cell viability decreased at concentrations of Frax1036 between 5 and 10 μM. Of the 4 agents we tested, STX2895 was the most robust for inducing apoptosis in NF2 tumour cell lines. PF and STX3451 displayed about the same potency, whilst Frax1036 was the least effective in blocking cell proliferation or causing apoptosis.

Next we investigated the mode of action of STX agents on the growth and/or induction of apoptosis in NF2 tumour cells and compared the effectiveness of these agents with those of Pak inhibitors. Our results, which will be discussed in the next section, indicated that STX agents caused depolymerization of microtubules, whilst Pak inhibitors did not have this effect.

Despite carefully repeated experiments (*n* = 5 [at least], on each of the cell lines) testing whether the STX agents act mechanistically on the same pathways on which we found them to be very effective on growth and apoptosis in human NF1 tumour cell lines, we currently have been able to detect NO significant effects of either of the agents on the growth, morphology of NF2 cell lines through the pathways that are known to be important for the growth of or cell death in NF1 human tumour cell lines (as detailed in Shen et al., 2015 [30]). This is an important point, because if the STX agents successfully reduce growth or induce apoptosis in NF2 cell lines as they do in NF1 cell lines, understanding the mechanism or mechanisms by which such effects occur is critical in order to plan the required preclinical animal studies that must follow the cell line studies if the agents are to be helpful eventually to both NF1 and NF2 patients.

### STX3451 and STX2895 cause nuclear fragmentation in NF2-null tumour cells

Our previous studies on NF1 cell lines demonstrated that STX3451 caused nuclear fragmentation in both the NF1-null malignant tumour cell line ST88 and in benign plexiform neurofibroma (PNF) cells, although this result was less significant for PNF cells [30].

**Fig. 4:**
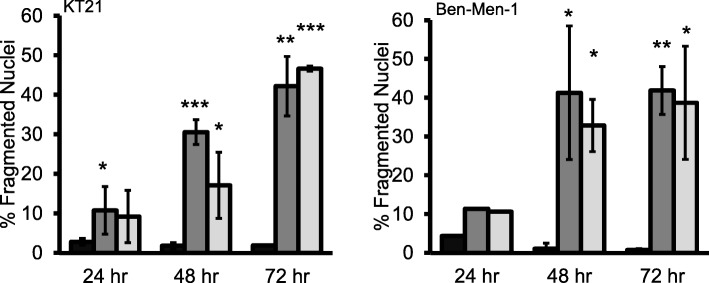
STX3451 and STX2895 caused nuclear fragmentation in both KT21 and Ben-Men-1 cells. DMSO control; STX3451; STX2895. Statistical significance: *p < 0.05; **p < 0.01; ***p < 0.005

We therefore examined whether this effect was also observed in our studies of NF2-null tumour cells. DAPI staining revealed that treatment with both STX3451 and STX2895 resulted in a dramatic increase in nuclear fragmentation, which was seen in both KT21 and Ben-Men-1 cell lines 48 h after treatment; with STX3451being more effective than STX2895 (Fig. 4). At 24 h, although STX3451 increased nuclear fragmentation in KT21 cells, the effect of STX2895 at this earlier time point was not significant, implying that most of the fragmentation caused by STX2895 occurred between 24 and 48 h after treatment. Further, nuclei fragmentation of KT21 was observed 72 h after treatment, with STX2895 more effective than STX3451, suggesting that STX2895 has a slower but consistent pace in causing fragmentation of nuclei.

For Ben-Men-1 cells, the dramatic increase seen in nuclei fragmentation also fell between 24 and 48 h after treatment, with STX3451 slightly more effective than STX2895. Neither analog caused significant increase of fragmentation between 48 and 72 h, presumably due to the greatly reduced cell viability seen during this time (Fig. 2). However, the nonsteroidal sulfamate derivatives of 2ME2 effectively promoted nuclear fragmentation in both NF1 [30] and NF2 null tumour cell lines.

### STX3451 and STX2895, but not the Pak inhibitors, induce microtubular disruption; treatment with all agents resulted in tumour cell apoptosis

STX3451 induced apoptosis and disrupted both microtubule- and microfilament-based cytoskeletal structures in NF1 ST88 cells, but did not induce programmed cell death in the benign PNF tumour cells [30]. To analyze apoptosis, we used an Annexin V antibody [51] to stain cells treated with STX agents and PAK inhibitors for 72 h. In healthy, quiescent cells phosphatidylserine (PS) is located exclusively at the inner cell membrane. Exposure of PS in a cell indicates early apoptosis [52]. Annexin V has a high binding affinity for PS in the presence of Ca^2+^ and is therefore commonly used to detect apoptotic cells.

**Fig. 5:**
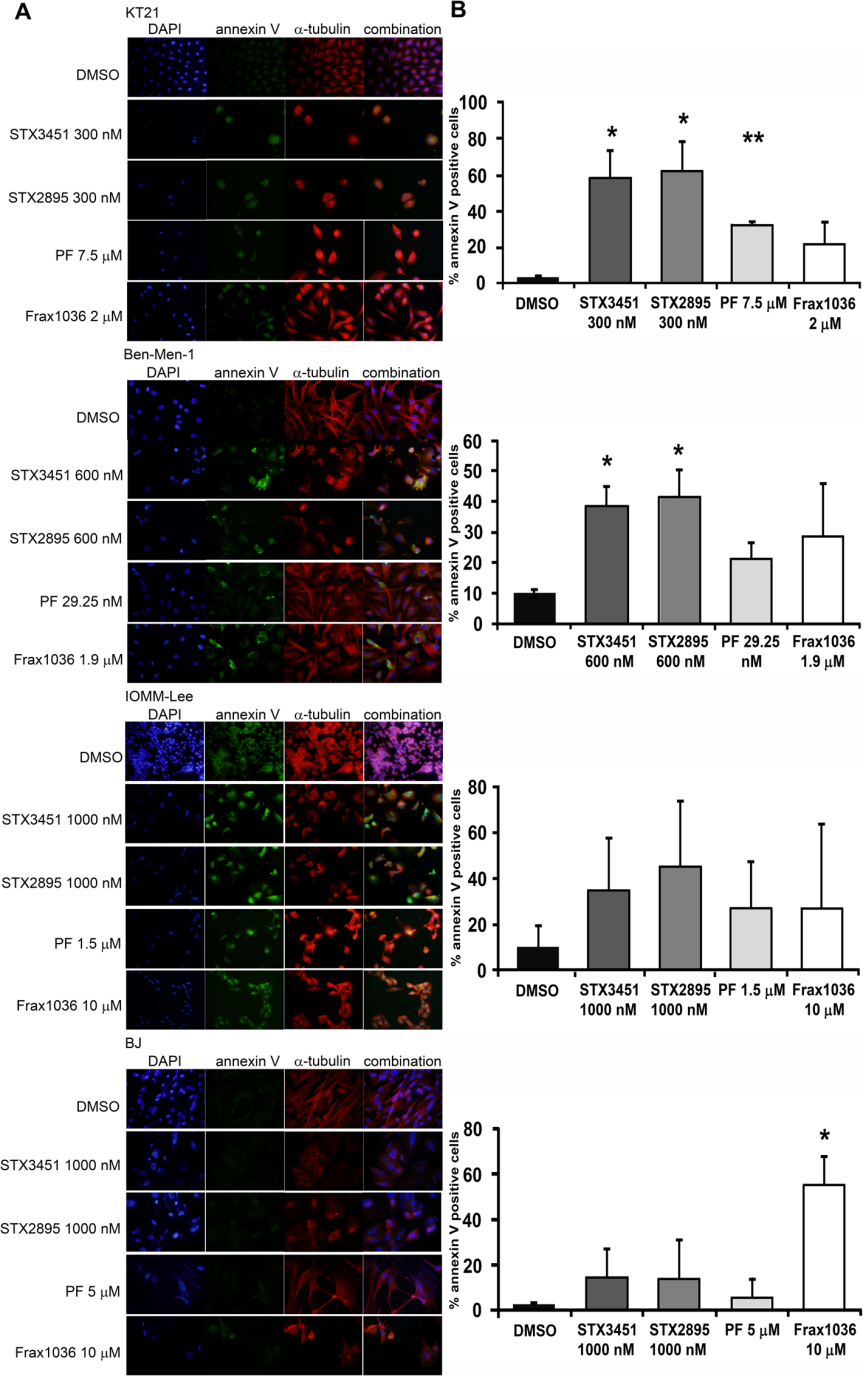
**a** STX3451 and STX2895 resulted in cytoskeletal structural changes and apoptotic cell death in NF2 tumor cells after 72 h of treatment. Cells were stained with DAPI for nuclei (blue), anti-α-tubulin antibody for microtubules (red), and annexin V for apoptotic cells (green). **b** Percentage of annexin-V-positive cells for control and treated conditions. DMSO; STX3451; STX2895; PF; Frax1036. Statistical significance: *p < 0.05; **p < 0.01

For KT21 cells, treatment with both STX3451 and STX2895 at 300 nM resulted in about 60% annexin V-positive-cells (Fig. 5). Even at much higher concentrations (7.5 μM and 2 μM, respectively), PF and Frax1036 resulted in only 30 and 20% of apoptotic KT21 cells at 72 h. Less than 10% of cells treated with the DMSO vehicle were annexin V-positive. Except for Frax1036, the increases seen in annexin V-positive cells are statistically significant.

For Ben-Men-1 cells, although cell viability was little affected after 4 days of treatment, about 40% of these cells treated with either STX3451 or STX2895 (at 600 nM) were apoptotic at 72 h, indicating that these analogues of 2ME2 also have effects on cell growth/apoptosis in the benign Ben-Men-1 cells. However, the effects seen are much less than those seen when either STX agent was used to treat malignant KT21 cells. By contrast, 72 h treatment with PF resulted in 20% annexin V-positive cells at very low concentration, the IC50 for KT21 was 29.25 nM [41]. Frax1036 at 1.9 μM caused 30% of Ben-Men-1 cells to become apoptotic at 72 h. Even though IOMM-Lee cell line viability was higher after 4 days, despite treatment with STX3451 or STX2895 at 1000 nM, annexin V-staining at 72 h demonstrated that 35 and 45% of these cells were undergoing cell death, respectively. The reason for this inconsistency is not clear. It could be that cells were still proliferating at earlier times, but eventually would be killed by higher concentrations of these 2ME2 analogs. Further studies are needed to determine the mechanism of action of these agents in IOMM-Lee cells. PF at 1.5 μM and Frax1036 at 10 μM resulted in apoptosis in about 25% of cells at 72 h, indicating that these agents are less effective in killing IOMM-Lee cells than either STX3451 or STX2895.

Finally, STX3451 and STX2895 at 1000 nM did not significantly increase the percentage of annexin V-positive BJ cells at 72 h, consistent with the results seen in the cell viability assays (discussed above). At concentrations that effectively induced apoptosis in NF2 tumour cells, STX3451 and STX2895 did not cause such programmed cell death in BJ fibroblasts. PF at 5 μM also did not increase the percentage of annexin V-positive BJ cells, whilst Frax1036 at 10 μM induced 55% cells to go through apoptosis.

STX3451 has been demonstrated to depolymerize microtubules in both NF1 ST88 and PNF cell lines [30]. Our current studies also confirmed the effect of STX3451 and STX2895 on microtubules in NF2-null tumour cells. Both agents caused cells to round up and to present with much shorter microtubules in all of the cell lines examined (Fig. 5a). Failure of cytokinesis was also seen in these treated cells, where cells with larger diameters were observed (Fig. 5a). PF and Frax1036, in contrast to the STX agents, did not result in microtubule disruption in most of the cell lines tested. A high concentration of Frax1036 (10 μM) resulted in rosette-like clusters of IOMM-Lee cells. However, the mechanism for this rosette formation is unknown.

Our results from annexin V and α-tubulin staining (Fig. 5) suggest that the two analogs of 2ME2 that we evaluated act differently from the Pak inhibitors on NF2 cells, especially in the effects seen on the microtubular cytoskeleton. However, both STX agents and Pak inhibitors can promote apoptosis in NF2 tumour cells.

### STX3451 and STX2895 decrease cell migration

**Fig. 6:**
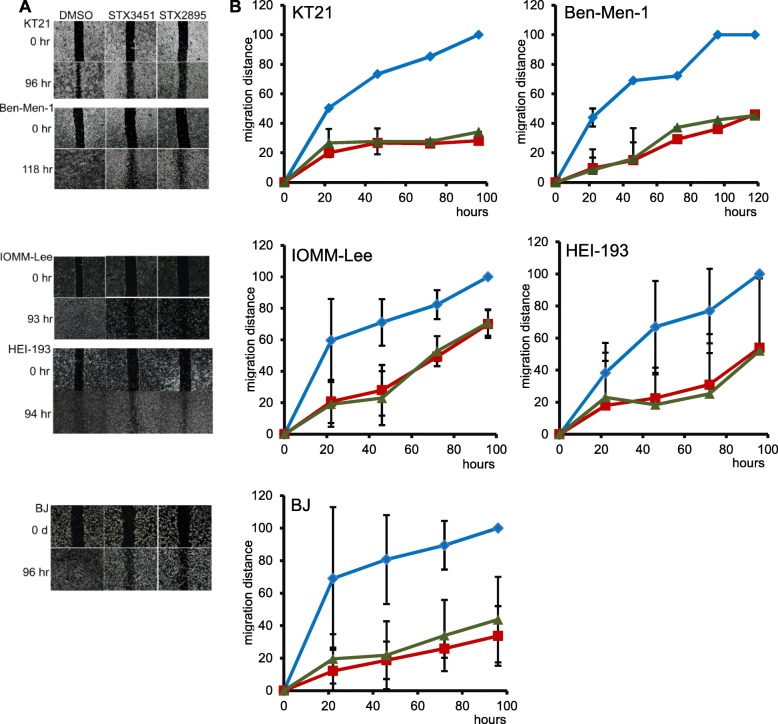
STX3451 and STX2895 treatment resulted in reduced cell migration of NF2 tumour cells and BJ cells after “wounding”. **a** Microscopic pictures of wound healing assays showing introduction of the wound (0 h) and at the end of the observation period (96–118 h after wound introduction). **b** Graphs show comparisons of the distances the cells moved from the wound periphery toward the middle of the open area at various time points. DMSO; STX3451; STX2895

PF and Frax1036 have been shown to inhibit invasiveness of both KT21 and Ben-Men-1 cells [41]. We, therefore, explored the possibility that STX3451 and/or STX2895 could also block cell migration in NF2-null tumour cells using “wound-healing” assays [49, 50] as we had previously done for NF1 cells [30]**.** We found that the cell lines, which we evaluated migrated to fill the wound gaps at different rates, and that STX3451 and STX2895 inhibited wound healing in the NF2 cells with different kinetics. The untreated control Ben-Men-1 cells were able to fill in the gaps by 4 days after the wound was made. However, cells treated with either STX3451 or STX2895 also filled in the gap, but at a much slower pace (Fig. 6). Control KT21 cells did not completely fill the gap even 4 days after the lesion was made, although the gap was almost eliminated by this time (Fig. 6a). KT21 cells treated with either STX3451 or STX2895 migrated to fill the gap at a much slower rate than the control cells and stopped migration entirely after 46 h. Both IOMM-Lee and HEI-193 cells closed the gap in DMSO-treated cultures (the control condition) after 4 days. However, STX3451 and STX2895 only slowed down the migration of these cells slightly for the first 2 days. The distance cells migrated to fill the gap by the third and fourth days was almost the same as that seen in the control cells treated with the DMSO vehicle, indicating that STX3451 and STX2895 did not inhibit cell migration over a period of 4 days, if this much time was allowed for recovery. Control BJ cells healed the wound gradually over the 96 h period, whilst cells treated with STX3451 and STX2895 migrated more slowly, and the gap was not filled in until 4 days after injury. Our results suggest that STX3451 and STX2895 are both effective in impeding the migration of KT21, Ben-Men-1, and BJ cells, but were less effective in preventing either IOMM-Lee or HEI-193 cells from migrating.

## Discussion

We previously demonstrated that STX3451 induces apoptosis in human NF1-deficient ST88 and S462 malignant peripheral nerve sheath tumour (MPNST) cell lines at very low concentration (300 nM), whilst arresting cell proliferation in an NF1^−/−^benign plexiform neurofibroma (PNF) [30]. In this study, using approaches and analyses identical to those we previously used in our NF1 tumour cell studies [30], we found that STX3451 and another potent sulfamoylated non-steroidal compound, STX2895, effectively induced cell death in NF2 deficient cell lines in vitro at concentrations between 0.3 μM and 1 μM. These two compounds have similar potencies in most of the NF2 cell lines, although STX2895 is slightly more potent than STX3451.

Intriguingly, our results showed that STX3451 and STX2895 were not as effective against a permanent NF2 wild type malignant meningioma cell line, IOMM-Lee, indicating that NF2 status could be important for the effects of these compounds. Our results also showed that these 2ME2 analogs were more potent than Pak inhibitors in inducing cell death. However, their mechanistic effects on the cells were different: STX3451 and STX2895 affect microtubules (Fig. 5), whilst Pak inhibitors act through the mTORC pathway and affect cell cycle progression by reducing β-catenin signaling, followed by reducing cyclin D1 expression [39]. Further, Pak inhibitors did not affect microtubule polymerization at the concentrations we used. Therefore, the 2ME2 analogs provide a new means of treating NF2-related disorders, and could potentially be combined with Pak inhibitors in multi-drug approach treatments.

We also found that STX3451 and STX2895 caused nuclear fragmentation and apoptosis in NF2 deficient cells, as shown by annexin V staining. We found that by 72 h after treatment with STX3451, the percentage of annexin V positive cells amongst the attached cells was 19 times greater in KT21 and 4 times greater in Ben-Men-1 cells than in control cells. Treatment with STX2895 resulted in 20 times more annexin V positive KT21 cells and 4.5 times more Ben-Men-1 cells than that of the controls. STX3451 and STX2895 also increased the percentage of annexin V positive IOMM-Lee cells, but this increase was not statistically significant, indicating that NF2 wild-type tumour cells are less sensitive to the 2ME2 analogs. In addition, as was also shown in our studies of NF1-deficient cells [30], STX3451 caused microtubule depolymerization in NF2-deficient cells. STX2895 had very similar effects to those of STX3451 in NF2-deficient cells. These effects on the cytoskeleton undoubtedly contribute to the decrease in cell migration (Fig. 6) after treatment with the 2ME2 analogs, since disrupting microtubules can affect cellular locomotion [53]. Both STX3451 [31] and STX2895 [32] have been shown to inhibit tubulin assembly in vitro, presumably by binding to the colchicine-binding site of tubulin. Indeed, our recent X-ray crystalographic study [54] has demonstrated, in atomic detail, that a non-steroidal quinazolinone sulfamate derivative, similar to those investigated here, can interact with the colchicine binding site and microtubule destabilization is likely achieved by preventing the curved-to-straight conformational transition in α/β-tubulin. This is the first atomic level demonstration of such an interaction for a sulfamate ester. Associated crystallographic work has also demonstrated the effectiveness of STX3451 in binding to the colchicine site [55].

Our previous study showed that the non-steroidal analog of 2ME2, STX3451, caused microtubule depolymerization and cell death in NF1 deficient tumour cells, even in the presence of elevated hormones [30]. Whether steroid hormones affect the growth of NF2^−/−^ vestibular schwannomas (VS) and/or meningiomas has not been well-studied. It has been shown that in sporadic VS, both oestrogen receptor (ER) and progesterone receptor (PR) were up-regulated, whilst in NF2-related VS, PR was down-regulated [56]. Amongst meningioma patients, two-thirds express PR (although − 30% express PR at low levels), and during malignant progression, PR expression tends to decrease [57]. PR-negative meningiomas also tend to be larger than those that are PR-positive [58], and expression of PR in meningiomas is correlated with a favourable prognosis [59]. However, several studies have shown that hormone replacement therapy in postmenopausal women is associated with increasing the risk of meningiomas [60, 61] and one case report showed that cessation of long-term use of the PR agonist megestrol acetate resulted in shrinkage of multiple meningiomas in one patient [62]. Treatment of progressive meningiomas with the PR antagonist mifepristone has not shown promising results [63]. Therefore, hormone therapies are not indicated and may even be contraindicated for NF2-related tumours. The ability of STX3451 and STX2895 to inhibit colchicine binding to microtubules could play a major role in microtubule depolymerization and subsequent death of NF2 cells. However, the fact that, even at high concentration as 1 μM, BJ and IOMM-Lee cells still proliferate, although at a slower pace than the controls (Fig. 2), indicates that these small molecules could interact with players in the Merlin pathway in addition to their effects on microtubules. These possibilities need to be further investigated.

Since Merlin affects several key signalling pathways in the cytoplasm and nucleus, including PI3K signaling, mTORC1, the RAS and Src pathways, the Hippo kinase cascade, and CRL4-DCAF [64], inhibitors of these pathways might serve as excellent candidates for drug targets. Indeed, of the targeted drugs tested in clinics, bevacizumab, which is a VEGF antibody, showed some improvement in hearing and tumour shrinkage in a quarter to a half of patients with VS, albeit in very small numbers of study subjects [23–25]. Some phase II clinical trials with bevacizumab are still ongoing. The results of treatments with everolimus, an mTOR inhibitor, showed that it stabilized tumours or delayed tumour growth [26], but was ineffective for progressive VS [65]. Lapatinib, a tyrosine kinase inhibitor for the epithelial growth factor receptor (EGFR), showed some success in reducing VS volumes and improving hearing responses in about one-quarter of a small group of patients in a phase II study [22], but no effect on meningiomas. Other phase II studies of this drug are also ongoing.

So far, none of the drugs tested in vitro or in pre-clinical or clinical trials has been effective on NF2 meningioma. Although, another mTOR inhibitor, AZD-2014, and a combination of smoothened receptor inhibitor, vismodegib, and FAK inhibitor, vistusertib, are in phase II trials. AR-42, a histone deacetylase inhibitor targeting CRL4-DCAF, which induced meningioma cell apoptosis in vitro and in xenografts, is also in phase I trials. Even if some inhibitors for NF2/Merlin signalling pathways have promising efficacy, drug resistance to these inhibitors may eventually occur and alternative choices are necessary. The microtubule-disrupting non-steroidal 2ME2 analogs could provide another option for treating NF2-related tumours, both vestibular Schwannomas and meningiomas, either alone, or in conjunction with other agents. Pre-clinical studies using several similar THIQ-based molecules have been shown to be safe and efficacious for reducing the size of human breast cancer [66] and melanoma xenografts [32, 45] in immunocompromised mice. Before testing the usefulness of STX3451 and STX2895 in human schwannoma and meningioma patients, pre-clinical studies are warranted. It is our hope that someday these small molecules can advance to therapeutic use in such patients.

## Conclusion

Both STX3451 and STX2895 provide new approaches for inducing cell death and lowering tumour burden in NF2 as well as in NF1, which both have limited treatment options.

## Data Availability

All data generated or analyzed during this study are included in this published article. Extra information can be obtained from the corresponding author if needed.

## Abbreviations

ER: oestrogen receptor
Merlin: Moesin-Ezrin-Radixin-like protein
MPNST: malignant peripheral nerve sheath tumours
NF1: Neurofibromatosis 1
NF2: Neurofibromatosis 2
PNF: plexiform neurofibroma
PR: progesterone receptor
THIQ: tetrahydroisoquinoline
VS: vestibular schwannomas
